# LncRNA *BCLET* variant confers bladder cancer susceptibility through alternative splicing of *MSANTD2* exon 1

**DOI:** 10.1002/cam4.6072

**Published:** 2023-05-21

**Authors:** Hanting Liu, Xi Wang, Zheng Guo, Guanting Sun, Qiang Lv, Chao Qin, Lin Yuan, Yunyan Wang, Mulong Du, Meilin Wang, Zhengdong Zhang, Haiyan Chu

**Affiliations:** ^1^ Department of Environmental Genomics, Jiangsu Key Laboratory of Cancer Biomarkers, Prevention and Treatment, Collaborative Innovation Center for Cancer Personalized Medicine, School of Public Health Nanjing Medical University Nanjing China; ^2^ Department of Genetic Toxicology, The Key Laboratory of Modern Toxicology of Ministry of Education, Center for Global Health, School of Public Health Nanjing Medical University Nanjing China; ^3^ Department of Urology The First Affiliated Hospital of Nanjing Medical University Nanjing China; ^4^ Department of Urology Jiangsu Province Hospital of Traditional Chinese Medicine Nanjing China; ^5^ Department of Urology The Affiliated Huai'an First People's Hospital of Nanjing Medical University Nanjing China

**Keywords:** alternative splicing, bladder cancer, genetic variations, lncRNA *BCLET*, molecular mechanism

## Abstract

**Background:**

Alternative splicing (AS)‐related single nucleotide polymorphisms (SNPs) are associated with risk of cancers, but the potential mechanism has not been fully elucidated.

**Methods:**

Two‐stage case–control studies comprising 1630 cases and 2504 controls were conducted to investigate the association between the AS‐SNPs and bladder cancer susceptibility. A series of assays were used to evaluate the functional effect of AS‐SNPs on bladder cancer risk.

**Results:**

We observed that SNP rs558814 A>G located in lncRNA *BCLET* (*Bladder Cancer Low‐Expressed Transcript,* ENSG00000245498) can decrease the risk of bladder cancer (odds ratio [OR] = 0.84, 95% confidence interval [CI] = 0.76–0.92, *p* = 3.26 × 10^−4^). Additionally, the G allele of rs558814 had transcriptional regulatory effects and facilitated the expression of *BCLET* transcripts, including *BCLET*‐long and *BCLET*‐short. We also found decreased *BCLET* expression in bladder cancer tissues and cells, and *BCLET* transcript upregulation substantially inhibited tumor growth of both bladder cancer cells and xenograft models. Mechanistically, *BCLET* recognized and regulated AS of *MSANTD2* to participate in bladder carcinogenesis, preferentially promoting the production of *MSANTD2‐004*.

**Conclusions:**

SNP rs558814 was associated with the expression of *BCLET*, which mainly increased the expression of *MSANTD2‐004* through AS of *MSANTD2*.

## 1 INTRODUCTION

With approximately 81,180 new cases and 17,100 deaths estimated globally, and incidence rates approximately three times higher among men than among women, bladder cancer is the tenth most common malignancy worldwide.^1^ In China, bladder cancer is one of the most common tumors of the male urinary system.^2^ Cigarette smoking and occupational exposure are the main risk factors for bladder cancer, with almost 50% of new cases attributed to smoking in both sexes.^3^, ^4^, ^5^ Prospective twin studies also showed that genetic factors contribute to 30% of the risk of bladder cancer development.^6^ Genome‐wide association studies (GWASs) have identified numerous single nucleotide polymorphisms (SNPs) associated with bladder cancer risk.^7^, ^8^ Identifying the underlying molecular mechanism of these risk SNPs has provided new opportunities to elucidate the etiology of bladder tumorigenesis.^9^, ^10^

The removal of introns is a crucial step in the process of pre‐mRNA splicing that joins exons together to generate mature mRNA products.^11^ Alternative splicing (AS) is a ubiquitous post‐transcriptional regulatory mechanism of eukaryotic gene expression that allows the production of multiple mRNA species with distinct activities from a single gene.^12^ Genome‐wide high‐throughput sequencing methods have estimated that approximately 95% of multiexon genes undergo AS events in the human transcriptome.^13^ Studies have shown that there are only approximately 20,000 protein coding genes in the human genome, and the number of unique mRNA isoforms generated from genes may exceed this number by 10 times.^14^ AS of pre‐mRNAs can dramatically increase transcriptomic and proteomic diversity through forms that include cassette exon skipping, alternative 5′ and 3′ splice sites, intron retention, mutually exclusive exons, and more complex AS patterns.^13^, ^15^ These splicing mRNA isoforms may have distinct regulatory properties, such as effects on mRNA stability, localization, and translational efficiency, and can generate different protein isoforms with diverse structures and functions.^16^, ^17^

Studies have found that AS regulates gene expression in a cell type‐specific and developmental stage‐specific manner and is essential for normal biological processes.^18^ In addition, aberrant AS events of mRNAs play an important role in the occurrence and development of many diseases, including cancers.^19^, ^20^, ^21^ Whole‐genome studies have found that SNPs in the mRNA splicing sequence of the human genome could affect RNA splicing and contribute to many diseases.^22^ In a recent study of prostate cancer, the risk SNP rs11672691 mediated the expression of different lncRNA *PCAT19* splicing isoforms through promoter‐to‐enhancer switching.^23^ Clearly, AS‐related SNPs (AS‐SNPs) can provide a primary link between genetic variation and cancer risk.^24^, ^25^

In this study, we systematically screened AS‐SNPs in the single nucleotide polymorphism database (dbSNP), evaluated the effects of AS‐SNPs on bladder cancer risk in Chinese populations, and explored the genetic and epigenetic biological effects of AS‐SNPs in bladder tumorigenesis.

## 2 METHODS

### 2.1 Study subjects

The genotyping data of 580 bladder cancer cases and 1101 controls were used in the discovery stage.^26^ In the validation study, we enrolled 1050 cases and 1403 controls.^27^ All cases were pathologically diagnosed as patients with bladder cancer who had not received radiotherapy or chemotherapy. The controls were from those undergoing a health check‐up in the same geographical region and matched with the age (±5 years) and gender frequency of the cases. All subjects in this study signed an informed consent form, and the study was approved by the Ethics Committee of Nanjing Medical University (2018‐617). The details for subject recruitment are provided in the Supplementary Materials and Methods, and the demographic characteristics of all subjects are shown in Table S1.

### 2.2 Selection of AS‐SNPs

The dbSNP database was downloaded to obtain the location and functional annotations of all SNPs in the genome, and a total of 108,049 SNPs with annotation information of “splice‐3” or “splice‐5” were extracted. Of these SNPs, 1518 SNPs were genotyped or input in our Illumina chips. The inclusion criteria were as follows: minor allele frequency (MAF) >0.05, call rate of genotypes >95%, and *p* > 0.05 for Hardy–Weinberg equilibrium (HWE). Eventually, a total of 206 variants were enrolled for further analysis.

### 2.3 Screening and functional prediction for candidate AS‐SNPs and genes

The functional scores for candidate AS‐SNPs were calculated through RegulomeDB, HaploReg v4, SNPinfo Web Server and CancerSpliceQTL. Regulatory scores for candidate SNPs were provided for candidate AS‐SNPs, including the items of histone modification, transcription factor binding, expression quantitative trait loci (eQTL), and splicing quantitative trait loci (sQTL). The details for functional prediction are provided in the Supplementary Materials and Methods.

The genomic sequences and locations of gene transcripts were obtained from the Ensembl genome. The Genotype‐Tissue Expression project was used to study the expression levels of different gene transcripts in bladder tissue. The protein coding ability of lncRNAs was evaluated through the Coding Potential Assessment Tool (CPAT). Another dataset with 384 bladder cancer tissues and 19 normal tissues from The Cancer Genome Atlas (TCGA) allowed us to analyze the eQTL effect of candidate SNPs as well as the expression difference of genes in bladder cancer tissues and adjacent tissues. Kaplan–Meier survival analysis with a log‐rank *p‐*value and hazard ratio based on *MSANTD2* expression was obtained using Kaplan–Meier Plotter. Uniform Resource Locators of the online bioinformatics database are described in the Supplementary Materials and Methods.

### 2.4 RNA immunoprecipitation (RIP)

RIP assays were performed using a Magna RIP kit (Millipore) according to the manufacturer's recommendations. After cotransfection of pcDNA3.1‐BCLET‐long or pcDNA3.1‐BCLET‐short and pMS2‐GFP into bladder cancer cells for 24 h, the cells were collected, and RIP lysis buffer was added. Magnetic beads precoated with the target GFP antibody (Roche) or negative control antibody IgG were added to each sample, and the RNA was purified in the immune complexes. For the expression of *MSANTD2* bound by IgG antibody or GFP antibody, quantitative RT‐PCR was used, and the input was a self‐control.

### 2.5 Animal models

A total of 1 × 10^7^ T24 cells in 0.1 mL of PBS were stably transfected with *BCLET‐*long/*BCLET*‐short/NC lentiviral vector and then subcutaneously injected into the right flank of male nude mice (5 weeks old, six mice per group). Tumor growth was measured frequently. After 4 weeks, the mice were sacrificed, and tumor size and weight were examined. Hematoxylin and eosin staining of tumors was used to select representative areas, and immunohistochemical staining was utilized to observe the expression of the proliferation marker Ki67 (anti‐Ki67, ab15580, Abcam) and *MSANTD2*. All animal experiments were performed in accordance with the guidelines of the Institutional Animal Care and Use Committee of Nanjing Medical University for animal experiments (IACUC‐2005004).

### 2.6 Statistical analysis

The goodness‐of‐fit chi‐squared test was used to analyze whether the distribution of SNP genotypes in the control population met HWE. Student's *t*‐test and chi‐squared test were performed to compare the distribution of demographic characteristics in cases and controls. Multivariate logistic regression was conducted after adjustment for potential covariates, including age and sex, and odds ratios (ORs) and 95% confidence intervals (CIs) were calculated to determine the association between AS‐SNPs and bladder cancer risk. The two‐stage case–control study was combined using meta‐analysis to merge the effects of rs558814 on bladder cancer risk, with Cochran's *Q* test and statistical *I* ^2^ to assess heterogeneity between groups.

Student's *t‐*test and paired *t‐*test were performed to detect the difference between two groups and paired continuous variables, respectively, and analysis of variance was used to determine the difference among multiple groups of continuous variables. Pearson correlation analysis was conducted to evaluate the correlation between the two continuous variables. This study used PLINK 1.9 and SAS 9.2 statistical software for general statistical analysis. All tests were two‐sided tests, and *p* < 0.05 was considered statistically significant.

## 3 RESULTS

### 3.1 Study overview

**FIGURE 1:**
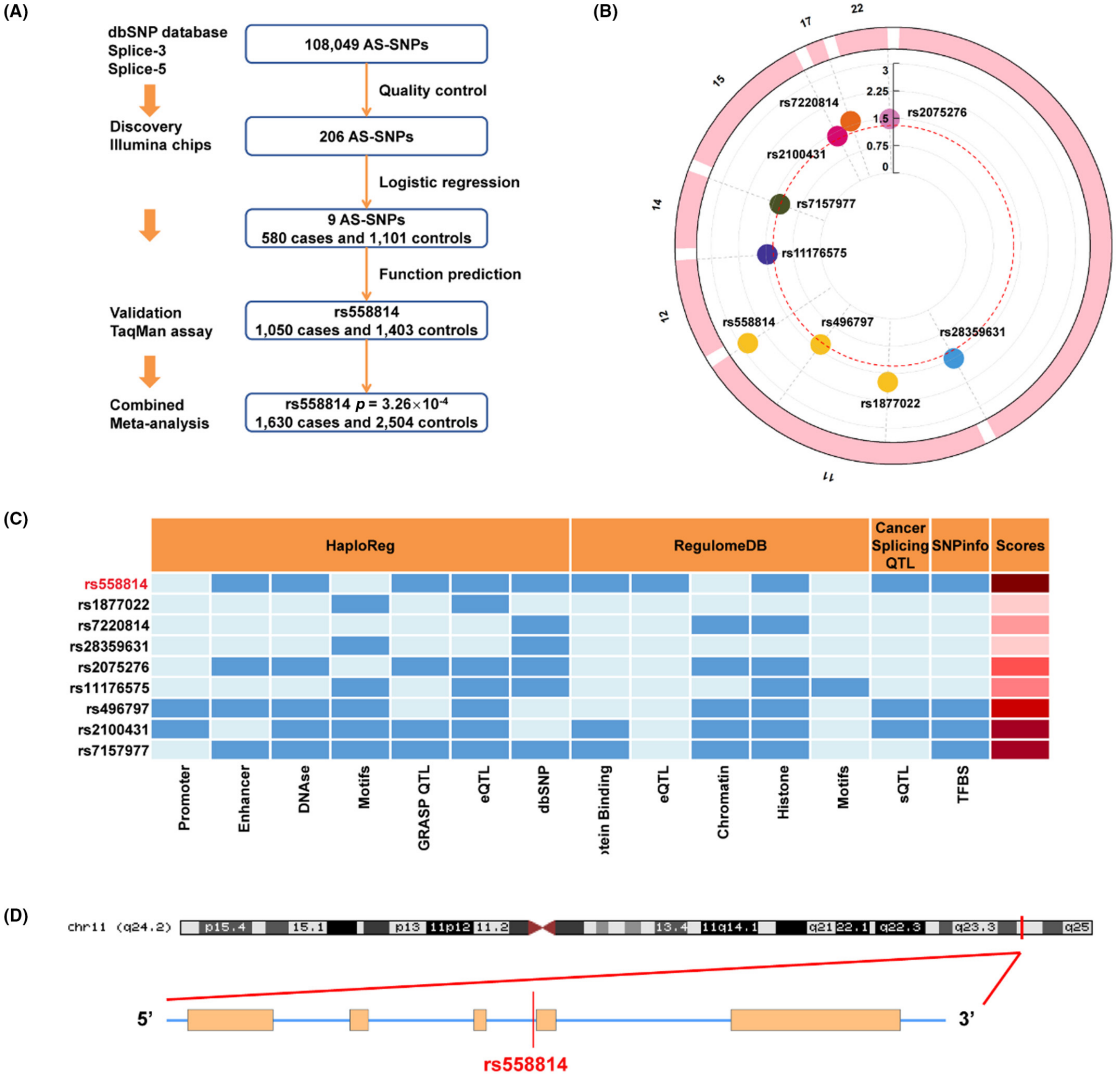
Alternative splicing‐single nucleotide polymorphism (AS‐SNP) selection and genetic variants associated with bladder cancer risk. (A) Flowchart of the AS‐SNP screen. (B) Circle Manhattan map of 9 AS‐SNPs. The ‐log10 *p‐*values of AS‐SNPs in the discovery stage according to their chromosomal positions. The red dotted circle indicates *p* < 0.05. (C) Functional annotation scores of nine candidate AS‐SNPs from RegulomeDB, HaploRefg, CancerSpliceQTL and SNPinfo. (D) SNP rs558814 at 11q24.2 is located in the intron of ENSG00000245498 (*RP1F1‐677M14.7*).

As shown in Figure 1A, AS‐SNPs with annotation information of “splice‐3” or “splice‐5” were screened from the dbSNP database, and previous Illumina chips were used for the genotyping of AS‐SNPs in bladder cancer cases and controls. In addition, the effect of AS‐SNPs on bladder cancer risk was calculated to identify risk SNPs, and the association was further validated in another independent population. Molecular assays were further used to explore the biological function of AS‐SNPs and genes involved in bladder cancer risk.

### 3.2 Identification of AS‐SNPs in bladder cancer risk

A total of 108,049 AS‐SNPs were extracted from the dbSNP database. After quality control including a call rate >95%, MAF >0.05, and *p* > 0.05 for HWE, we retained a total of 580 bladder cancer cases and 1,101 controls with 206 AS‐SNPs for further analysis (Figure 1A and Table S1). We identified nine AS‐SNPs significantly associated with bladder cancer susceptibility (*P* < 0.05, Figure 1B and Table S2). Functional annotation demonstrated that SNP rs558814 located at q24.2 on chr11 had the highest functional score and the strongest effect (Figure 1C,D). Thus, we selected SNP rs558814 as a candidate SNP for further investigation in bladder cancer.

We subsequently genotyped rs558814 in a validation study including 1,050 cases and 1,403 controls, and the characteristics of the subjects have been reported previously^27^ (Table S1 **)**. The results showed that rs558814 A>G decreased the bladder cancer risk in the additive model (OR = 0.88, 95% CI = 0.78–0.99, *p* = 0.033) and dominant model (OR = 0.84, 95% CI = 0.72–0.99, *p* = 0.037; Table S3). The combined analysis showed that rs558814 A>G was associated with a decreased risk of bladder cancer risk (OR = 0.84, 95% CI = 0.76–0.92, *p* = 3.26 × 10^−4^) with no heterogeneity (*I* ^2^ = 21.5%, *P*_het_ = 0.259; Table 1).

**TABLE 1:** SNP rs558814 A>G was associated with decreased bladder cancer risk.

Stages	Cases/controls	Genotypes (AA/AG/GG)		MAF (Cases/controls)	OR (95% CI)	*p*‐Value*
		Cases	Controls			
Discovery	580/1101	292/202/52	476/451/135	0.28/0.34	0.78 (0.67–0.91)	1.91 × 10^−3^
Validation	1050/1403	502/436/102	617/625/159	0.31/0.34	0.88 (0.78–0.99)	3.29 × 10^−2^
Combined	1630/2504	794/638/154	1093/1076/294	0.30/0.33	0.84 (0.76–0.92)	3.26 × 10^−4^

Abbreviations: CI, confidence interval; MAF, minor allele frequency; OR, odds ratio; SNP, single nucleotide polymorphism.

* *p‐*Values were calculated from logistic regression analysis adjusted for age and sex.

### 3.3 Effect of rs558814 on mediating lncRNA *BCLET* expression in bladder cancer

**FIGURE 2:**
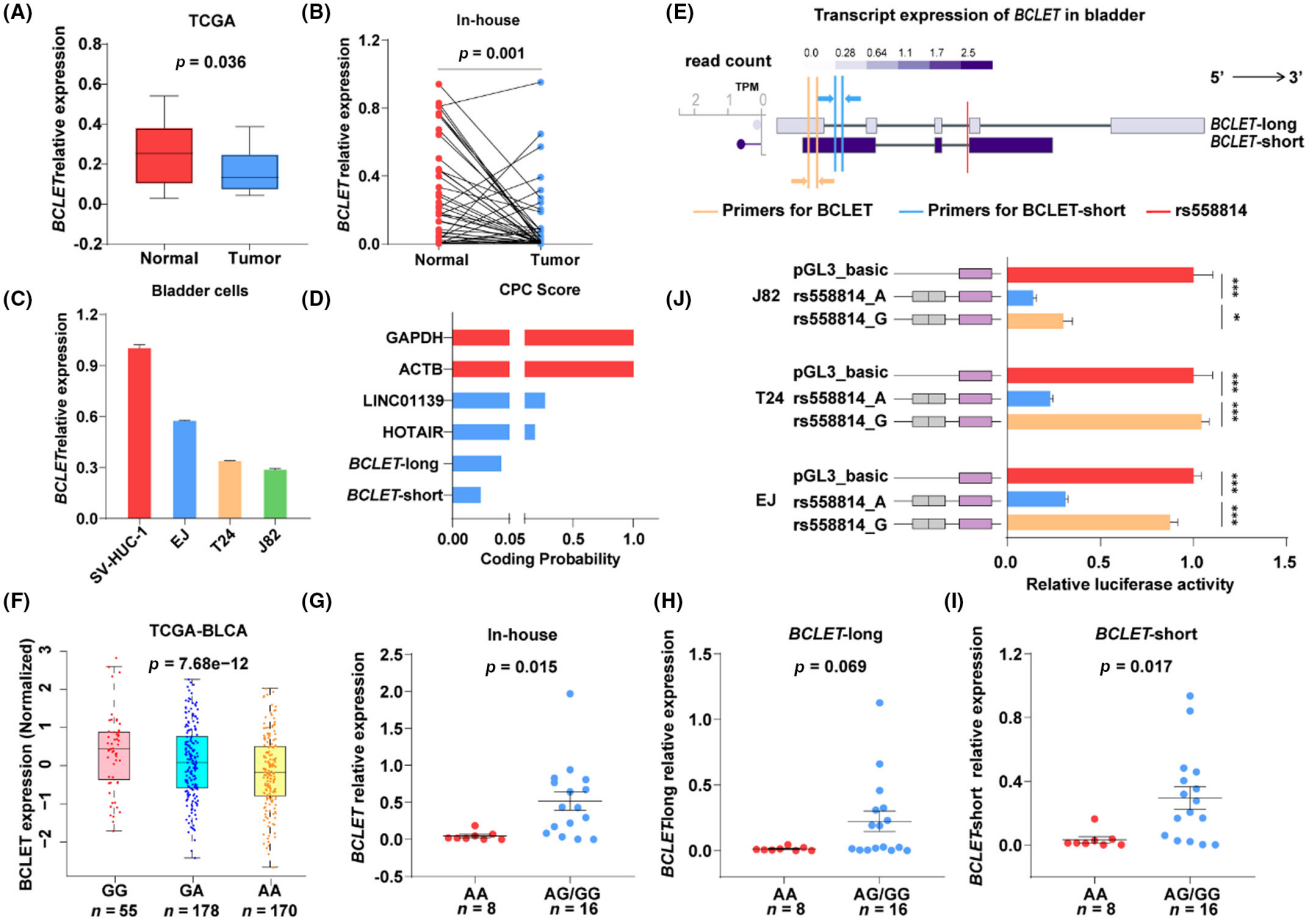
Single nucleotide polymorphism rs558814 regulated *BCLET* transcripts through transcriptional regulation. The expression of *BCLET* in The Cancer Genome Atlas (TCGA) data (A), 51 paired bladder cancer and adjacent normal tissues (B), and the bladder epithelial cell SV‐HUC‐1 and bladder cancer cells EJ, J82, and T24 (C). (D) The prediction of the protein coding ability of *BCLET*‐long and *BCLET*‐short by the CPC online tool. (E) Schematic diagram of the transcripts, expression and primers of *BCLET*. (F) The association between rs558814 genotypes and the expression of *BCLET* in TCGA bladder cancer database. (G‐I) The association between rs558814 genotypes and the expression of total *BCLET* (G), *BCLET*‐long (H) and *BCLET*‐short (I) in 24 bladder adjacent tissues. (J) The effect of rs558814 on the transcriptional activity of *BCLET* by luciferase reporter assays in bladder cancer cells.

According to Ensembl (GRCh37 version), rs558814 is located on the intron of ENSG00000245498 (RP11‐677M14.7), which is annotated as *MSANTD2* (Myb/SANT‐like DNA‐binding domain containing 2) antisense RNA and is generally categorized as an ncRNA. The public and in‐house dataset showed that the expression of *RP11‐677M14.7* was lower in bladder cancer tissues than in normal tissues (Figure 2A,B). A similar result was also found in bladder cell lines (Figure 2C). RP11‐677M14.7 coding probability prediction revealed extremely low predicted coding values by using the CPC score (Figure 2D). We further named this long ncRNA (lncRNA) RP11‐677M14.7 *BCLET* (*Bladder Cancer Low‐Expressed Transcript*). We further found different *BCLET* transcripts: *BCLET*‐long (ENST00000499143) and *BCLET*‐short (ENST00000529392) (Figure 2E). Quantitative analyses revealed that the distribution of *BCLET* in the nucleus accounted for more than 90% (Figure S1).

Subsequently, we explored the eQTL effect of the SNP rs558814 on *BCLET* transcript expression in bladder cancer. The results suggested that the rs558814 G allele was associated with a higher expression level of *BCLET* in bladder cancer tissues than the A allele (Figure 2F), and a similar effect was observed in in‐house bladder adjacent tissues (Figure 2G) and for *BCLET*‐short (Figure 2I). However, the *BCLET*‐long transcripts showed a trend without significant differences (Figure 2H). In addition, the luciferase reporter experiments revealed that compared with the A risk allele, the G allele was linked to increased *BCLET* transcriptional activity (Figure 2J). These results suggested that rs558814 may participate in bladder cancer risk by affecting the expression of *BCLET* through a transcriptional regulatory mechanism.

### 3.4 Biological function of lncRNA *BCLET* in the malignant progression

**FIGURE 3:**
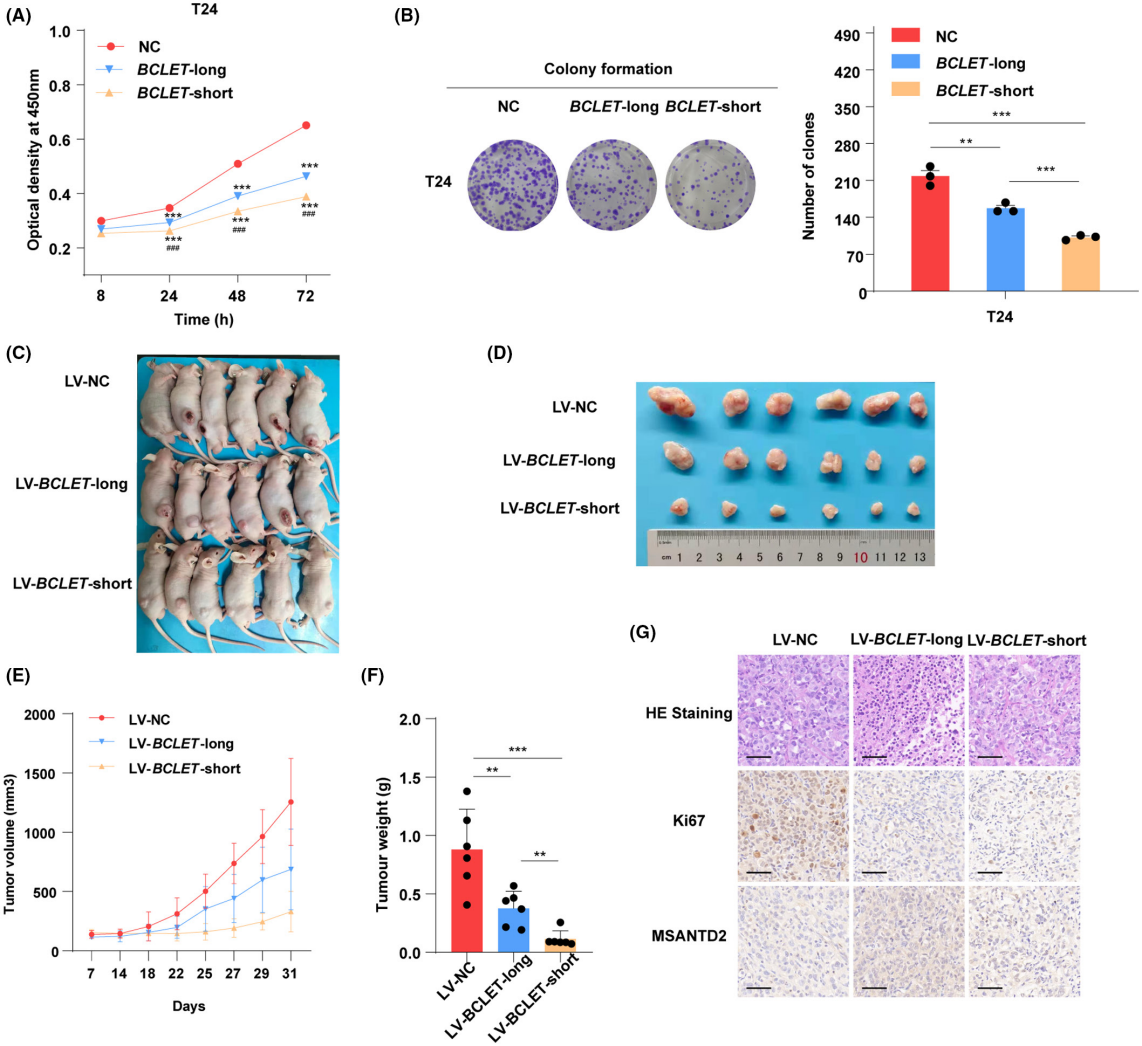
*BCLET* transcripts significantly inhibited tumor proliferation in vitro and in vivo. The effect of *BCLET* transcripts overexpression on cell viability was detected by CCK‐8 (A) and colony formation assays (B) in T24 cell lines. Tumor burdens after inoculation with LV‐*BCLET‐*long/LV‐*BCLET*‐short and LV‐NC were examined in nude mice (C) and their xenografts (D). (E) The tumor volumes were measured. (F) The tumor weights in the nude mice were determined after 4 weeks. (G) Representative images of HE and immunohistochemical staining for Ki67 and MSANTD2 in mouse tumors (scale bars = 50 μm).

We further explored the biological effect of *BCLET* transcripts on bladder cancer cell phenotypes. Overexpression of *BCLET*‐long or *BCLET*‐short (Figure S2) significantly suppressed the proliferation, clone formation, invasion, and migration of bladder cancer cells (Figure 3A,B; Figures S3 and S4A,B). In addition, *BCLET* transcripts increased bladder cancer cell apoptosis (Figure S4C). To further investigate the role of *BCLET* in bladder cancer in vivo, we stably transfected T24 cells with NC/*BCLET*‐long/*BCLET*‐short lentiviral vector. As shown in Figure S5, stable overexpression of *BCLET*‐long/*BCLET*‐short significantly suppressed the proliferation, clone formation, invasion, and migration of T24 cells. We then injected T24 cells with NC/*BCLET*‐long/*BCLET*‐short lentiviral vector into nude mice. Consistent with the in vitro cell phenotype, *BCLET* overexpression dramatically decreased the mean tumor weight and average tumor volume in mice (Figure 3C–F). As shown in Figure 3G, the tumor tissues overexpressing *BCLET*‐long/*BCLET*‐short showed significantly reduced expression of the proliferation marker Ki67 and notably increased expression of *MSANTD2*, a gene located near *BCLET*.

### 3.5 *BCLET* promotes AS of *MSANTD2* exon 1 in bladder cancer

**FIGURE 4:**
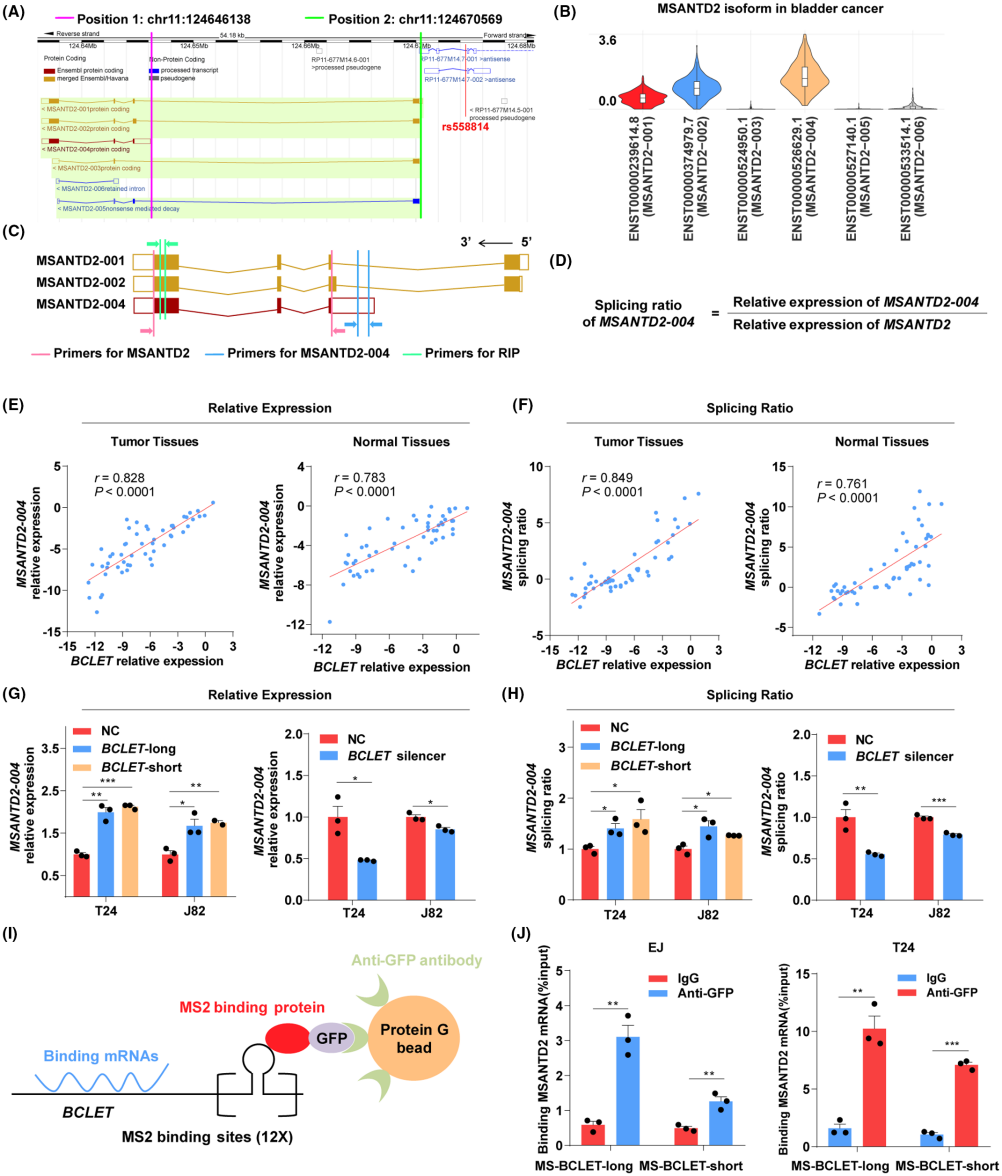
*BCLET* increased the expression level of the *MSANTD2‐004* isoform through alternative splicing of *MSANTD2* exon 1. (A) The location diagram of *BCLET* and *MSANTD2* transcripts. (B) Diagram and expression of the *MSANTD2* isoform in bladder cancer tissue. (C) Schematic diagram of primer design. (D) Calculation of the splicing ratio for *MSANTD2‐004*. The ratio of *MSANTD2‐004* expression to the total expression of *MSANTD2* was used to evaluate the splicing ratio of the *MSANTD2‐004* transcript, and the values were converted by log2. (E) The coexpression analysis of *BCLET* and *MSANTD2‐004* in bladder cancer tissues (left) and bladder adjacent tissues (right). (F) Correlation analysis between *BCLET* expression level and the *MSANTD2‐004* splicing ratio in bladder cancer tissues (left) and bladder adjacent tissues (right). The values were converted by log2. (G) The expression level of *MSANTD2‐004* in the bladder cancer cells transfected with the *BCLET* overexpression vector (left) and lncRNA Smart Silencer (right). (H) The splicing ratio of *MSANTD2‐004* in the bladder cancer cells transfected with the *BCLET* overexpression vector (left) and lncRNA Smart Silencer (right). (I) Schematic diagram of the RNA immunoprecipitation (RIP) experiment. (J) RIP experiments were used to investigate the relationship between *BCLET* and *MSANTD2* in bladder cancer cells.

Studies have shown that antisense RNA located in the nucleus can bind to its corresponding sense RNA to participate in the splicing regulation of sense RNA. In this study, *MSANTD2* is located at the 5′ end of *BCLET* and has 6 transcripts (Figure 4A). Among *MSANTD2* transcripts, *MSANTD2‐004* was found to have the highest expression level and lacked exon 1 (Figure 4B). We further designed primers and calculated the splicing rate of *MSANTD2‐004* to explore the splicing regulatory effect of *BCLET* on *MSANTD2* (Figure 4C,D). Coexpression analysis showed that *BCLET* and *MSANTD2‐004* had a significant positive correlation (*r* = 0.828 for bladder cancer tissues and 0.783 for bladder normal tissues, *p* < 0.0001; Figure 4E). In addition, a novel positive correlation was found between the expression of *BCLET* and the splicing ratio of *MSANTD2‐004* (*r* = 0.849 for bladder cancer tissues and 0.761 for normal bladder tissues, respectively, *p* < 0.0001; Figure 4F). We further found that the overexpression of *BCLET*‐long or *BCLET*‐short significantly increased the expression level and splicing ratio of *MSANTD2‐004*, and low expression of *MSANTD2‐004* and a low splicing ratio were observed with decreasing expression of *BCLET* in bladder cancer cells (Figure 4G,H and Figure S6). RIP experiments were conducted to investigate the relationship between *BCLET* and *MSANTD2* mRNA (Figure 4I). *MSANTD2* enriched by GFP antibody significantly increased compared with that of the negative control, suggesting binding between lncRNA *BCLET* and *MSANTD2* (Figure 4J). These results suggested that as the antisense RNA of *MSANTD2*, lncRNA *BCLET* may bind to *MSANTD2* mRNA to preferentially promote the expression of *MSANTD2‐004*.

### 3.6 Effect of *MSANTD2* on malignant bladder cancer cell phenotypes

**FIGURE 5:**
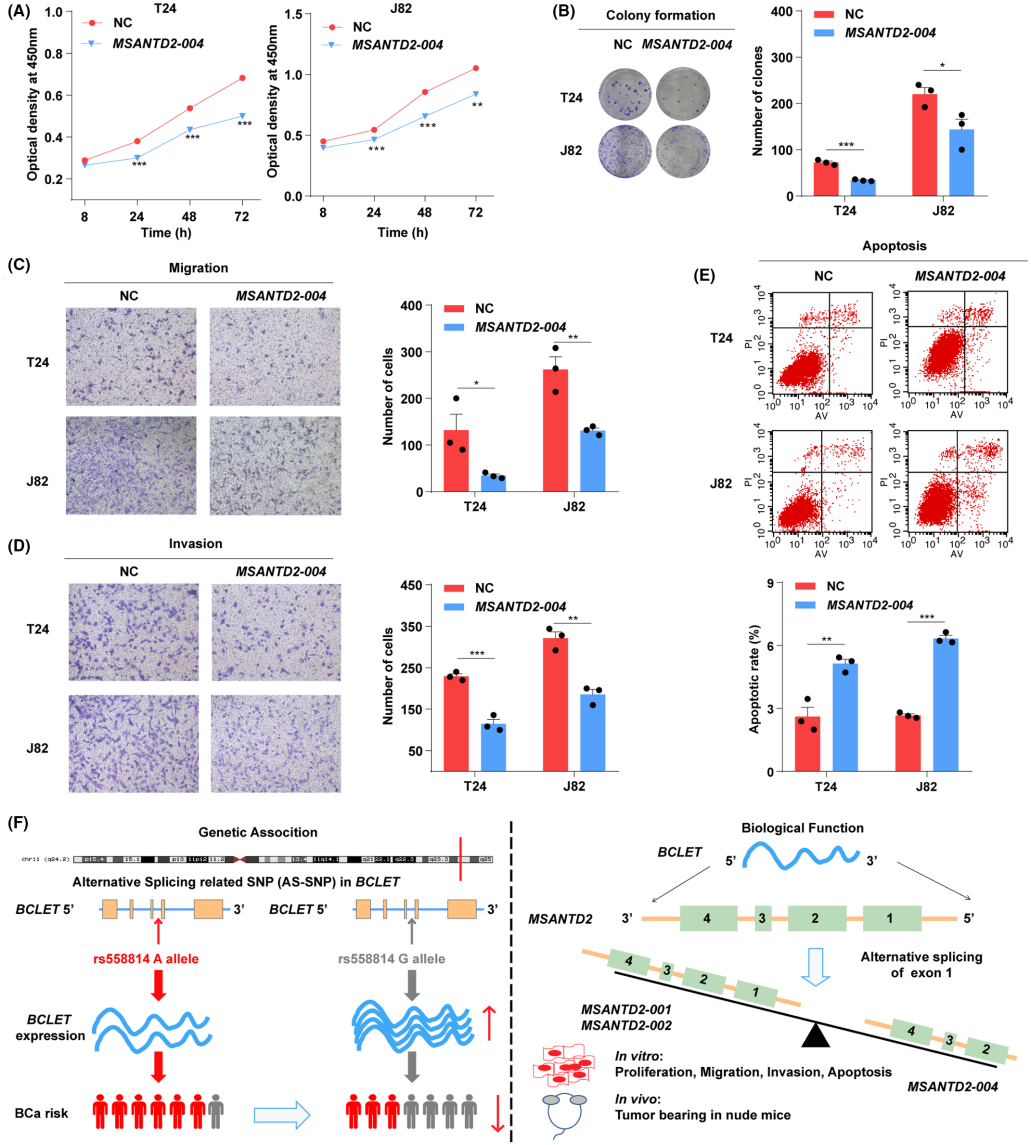
MSANTD2‐004 regulated the bladder cancer cells malignant phenotype. (A) CCK8 assay. (B) Cell clone formation. (C) Cell migration. (D) Cell invasion. (E) Cell apoptosis. (F) Graphical representation of the regulation and function of *BCLET* rs558814 in bladder cancer. Single nucleotide polymorphism (SNP) rs558814 A>G in *BCLET* was significantly associated with a reduced risk of bladder cancer. Furthermore, *BCLET* mediated cell proliferation, Transwell activity, and tumorigenesis to participate in bladder oncogenesis. *BCLET* ultimately bound *MSANTD2* and increased the expression of *MSANTD2‐004* by modulating alternative splicing (AS) of *MSANTD2* exon 1.

We further explored the biological function of the splicing isoform *MSANTD2‐004* in bladder cancer. After overexpression of *MSANTD2‐004* in bladder cancer cells (Figure S7), we found that *MSANTD2‐004* was involved in decreased bladder cell proliferation, plate cloning, invasion and migration and increased the number of apoptotic cells (Figure 5A–E). Taken together, the above results suggested that *MSANTD2‐004* may play a tumor suppressor gene‐like role in bladder carcinogenesis.

## 4 DISCUSSION

GWASs have become a powerful tool to explore the etiology of complex diseases, and many genetic variants for bladder cancer have been discovered.^8^ However, GWAS‐identified loci can only explain part of the etiology of bladder cancer, and functional studies of these loci in carcinogenesis are relatively scarce.^28^ Emerging evidence has demonstrated that the splicing regulatory effects of genetic variants can mediate gene expression, which provides a novel perspective on functional SNPs and biological mechanisms.^24^, ^29^ In this study, two‐stage case–control studies showed that the SNP rs558814 affected the expression of lncRNA *BCLET* through regulation of transcriptional activity; *BCLET* altered the splicing pattern of *MSANTD2* mRNA to promote the expression of *MSANTD2‐004*, which was involved in bladder carcinogenesis (Figure 5F).

AS events are key processes for cell development, differentiation and functional regulation, which can make a precursor RNA produce multiple mRNA splicing isoforms.^30^, ^31^, ^32^ Abnormal changes in AS events of key tumor‐related genes can affect gene expression and lead to tumorigenesis.^33^, ^34^ Emerging evidence has confirmed that multiple splicing isoforms produced by AS events may have different or even opposite biological effects.^31^, ^35^ For modulation of apoptosis, AS of the *BCL‐X* gene produces a proapoptotic *BCL‐XS* splice variant and an antiapoptotic *BCL‐XL* variant, which balance the cell apoptosis pattern in the development of several diseases and tumorigenesis.^36^

AS‐SNPs were reported to be potentially pathogenic variants that may participate in the occurrence and development of diseases, and functional annotation has also shown that risk SNPs are significantly enriched in AS sites and splicing factor gene coding regions.^29^ With genotype data and corresponding AS values from TCGA, thousands of sQTLs across multiple cancer types were identified, further elucidating the modulation of AS events by genetic variants.^37^, ^38^ LncRNA *BCLET* is the antisense RNA of *MSANTD2*, which is also known as C11orf61 and is significantly enriched in autism‐related postzygotic mutations in whole‐exome sequences.^39^ After overexpression of *MSANTD2* or lncRNA *BCLET*, we found that the malignant phenotype of bladder cancer cells was suppressed and tumor growth was inhibited in vivo, suggesting that *MSANTD2* and lncRNA *BCLET* may act as tumor suppressors in bladder cancer and may be used as a therapeutic target in the future.

Studies have reported that AS events are usually regulated by cis‐acting elements and trans‐acting factors. LncRNAs may regulate the splicing of target genes by binding to enhancing splicing factors to promote the inclusion of exons and interacting with silencing splicing factors to promote exon skipping.^40^ In addition, the antisense RNA located in the nucleus may bind to its corresponding sense RNA, which can mask the splicing site of the coding gene, thereby changing the balance between splicing isoforms.^41^ For instance, RevErbAα, the antisense RNA of thyroid hormone receptor α (TRα), can bind to the mRNA of *TRα1* and *TRα2* to modulate the splicing process of *TRα*.^42^ In addition, it was demonstrated that antisense RNA lncRNA *UXT‐AS* could mediate colorectal cancer progression by reducing the *UXT1* transcript, which could increase cell apoptosis, and upregulating the *UXT2* transcript, which enhanced cell proliferation.^43^

In this study, we found that the expression of the lncRNA *BCLET* and the splicing ratio of *MSANTD2* were significantly positively correlated. After overexpression or interference with *BCLET* in cells, the expression and splicing ratio of *MSANTD2* changed accordingly, which further confirmed the splicing effect of lncRNA *BCLET* on *MSANTD2*. The RIP experiment in this study suggested that lncRNA *BCLET* may bind to *MSANTD2*, thereby regulating the AS and expression of *MSANTD2*. However, the lncRNA *BCLET* may also interact with splicing factors to regulate the splicing of *MSANTD2*, but the mechanism needs to be further explored.

## 5 CONCLUSIONS

We demonstrated that rs558814 is a novel bladder cancer susceptibility locus that modulates *BCLET* expression by altering transcriptional activity. LncRNA *BCLET* is involved in tumorigenesis by suppressing the malignant phenotype of bladder cancer through AS of *MSANTD2* to preferentially produce the isoform *MSANTD2‐004*. This study provides important insight into the mechanism of genetic susceptibility to bladder cancer and establishes a basis for elucidating the etiology and biological mechanism of bladder cancer.

## AUTHOR CONTRIBUTIONS

**Hanting Liu:** Conceptualization (equal); formal analysis (equal); writing – original draft (equal). **Xi Wang:** Data curation (equal); formal analysis (equal); investigation (equal). **Zheng Guo:** Data curation (equal); formal analysis (equal); investigation (equal). **Guanting Sun:** Data curation (equal); formal analysis (equal); investigation (equal). **Qiang Lv:** Formal analysis (equal). **Chao Qin:** Formal analysis (equal). **LIn Yuan:** Formal analysis (equal). **Yunyan Wang:** Formal analysis (equal). **Mulong Du:** Formal analysis (equal); methodology (equal); software (equal); validation (equal). **Meilin Wang:** Formal analysis (equal); methodology (equal); software (equal); validation (equal). **Zhengdong Zhang:** Formal analysis (equal); methodology (equal); software (equal); validation (equal). **Haiyan Chu:** Conceptualization (equal); funding acquisition (equal); writing – review and editing (equal).

## FUNDING INFORMATION

This study was supported in part by the National Natural Science Foundation of China (grant 81872691), Collaborative Innovation Center for Cancer Personalized Medicine and Priority Academic Program Development of Jiangsu Higher Education Institutions (Public Health and Preventive Medicine).

## CONFLICT OF INTEREST STATEMENT

No potential conflicts of interest are disclosed.

## Supporting information


Data S1.

## Data Availability

The data that support the findings of this study are available upon reasonable request from our group but restrictions apply to the availability of these data, which were used under Nanjing Medical University approval and were approved by Nanjing Medical University authority protecting privacy and personal data for the current study, and so are not publicly available. Further information is available from the corresponding author upon request.
