# Supplementary material for: LncRNA BCLET variant confers bladder cancer susceptibility through alternative splicing of MSANTD2 exon 1
Source: Cancer Med. 2023 May 21;12(13):14440–51. doi: 10.1002/cam4.6072 (PMC10358201; doi:10.1002/cam4.6072)
Supplement: Supplementary file 1 — Data S1. [file CAM4-12-14440-s001.docx]

**Supplementary materials and methods**

**Study subjects**

In the discovery stage, 580 bladder cancer cases were recruited from Nanjing (China) starting in May 2003, and 1,101 controls were selected from the same geographical region. The validation stage included 1,050 cases, and 1,403 controls were mainly recruited from the First Affiliated Hospital and Huai-An Affiliated Hospital of Nanjing Medical University between January 2003 and May 2013. The demographic characteristics of all individuals are shown in **Table S1**.

**Screening and functional prediction for AS-SNPs**

The scoring criteria are as follows: For HaploReg v4.1 and SNPinfo Web Server, the SNP function score is the sum of the functional annotation items; for the RegulomeDB database, the SNP function score is the reverse score of the database; for the CancerSplicingQTL, only SNPs with an sQTL effects score of one point were used; the sum of the four scores is the total score for each candidate SNP.

**SNP genotyping**

Genotyping of the discovery stage was conducted using an Illumina Human Omni ZhongHua Bead chip and HumanOmniExpress chip. Genotyping for rs558814 in the validation stages was conducted using TaqMan assays (Applied Biosystems). Blinding of technicians was used to control the quality of the genotyping process. The sequences of the primers and probes for TaqMan are shown in **Table S4**.

**Patient sample collection and extraction of DNA and RNA**

A total of 51 pairs of bladder cancer tissues with 24 peripheral blood samples were collected in this study. All samples were originally collected from patients with bladder cancer undergoing surgery in Jiangsu Province Hospital of Traditional Chinese Medicine and The First Affiliated Hospital of Nanjing Medical University. Blood samples of each subject were collected to extract genomic DNA using a DNA extraction kit (Tiangen), and total RNA was collected using TRIzol Reagent (Invitrogen) from tissues according to the manufacturers’ protocols.

**Cell culture and isolation of cytoplasmic and nuclear RNA**

The human bladder cancer cell lines (EJ, T24 and J82) and bladder epithelial cell line (SV-HUC-1) were used for assays. All cell lines were purchased from the Shanghai Institute of Biochemistry and Cell Biology, Chinese Academy of Sciences (Shanghai, China). Among these cells, SV-HUC-1, EJ and T24 cells were cultured with 1640 medium (Biological Industries) containing 10% FBS (Gibco), 100 U/ml penicillin (Gibco) and 100 μg/ml streptomycin (Gibco), and J82 cells were cultured with MEM (KeyGEN). All cells were cultured at 37°C in a 5% CO_2_ atmosphere in a humidified incubator. Total RNA was collected from the parental and infected cells using TRIzol Reagent (Invitrogen) according the manufacturers’ protocols. In addition, cytoplasmic and nuclear RNA were extracted from cells and purified using a Protein and RNA Isolation Kit (Thermo Fisher) according to the manufacturer’s protocols.

**Authentication of cell lines**

All cell lines have been authenticated using short tandem repeat profiling within the last 2 years.

**Quantitative RT‒PCR (qRT‒PCR)**

After isolation of RNA, total RNA was reverse transcribed into cDNA with a High-Capacity cDNA Reverse Transcription Kit (Invitrogen) according to the manufacturer’s protocols, and the cDNA templates were used to quantify the expression of lncRNA *BCLET* and *MSANTD2*. Quantitative RT‒PCR (qPCR) was conducted with a SYBR Green qPCR system (Vazyme) using a LightCycler 480 or LightCycler 96 Real-Time PCR System (Roche). *GAPDH* or *U6* was used as an endogenous control for cytoplasmic and nuclear RNA expression, respectively. The comparative Ct method was used to calculate the relative expression of RNA or transcripts. The primers were synthesized by Realgene, and the sequences are presented in **Table S4**. Because the specific primers of the *BCLET*-long transcript were difficult to design, the expression of *BCLET*-long was calculated by the total expression of *BCLET,* removing the expression of *BCLET*-short in this study.

**Construction of luciferase plasmids and luciferase reporter assays**

For the detection of the transcriptional activity of rs558814, the lncRNA *BCLET* promoter containing the rs558814 A or G allele was synthesized into the pGL3-basic vector by GENEray. Sanger sequencing was used to verify the sequence. Lipofectamine 3000 (Thermo Fisher) was used to cotransfect the constructed luciferase reporter gene plasmid and internal reference PRL-SV40 plasmid (GENEray) into the three bladder cancer cell lines (EJ, J82 and T24) according to the manufacturer's instructions. Approximately 24 h after transfection, the luciferase activity of the cells was detected using a dual-luciferase assay system (Promega). The ratio of the fluorescence value of firefly to the fluorescence value of Renilla was calculated to compare the difference in transcriptional activity of plasmids carrying different alleles.

**Construction of overexpression plasmids and siRNAs**

For the overexpression of *BCLET*-long, *BCLET*-short, and *MSANTD2-004*, three transcript sequences were synthesized and subcloned into the pcDNA3.1 vector (GENEray). For the knockdown of lncRNA *BCLET*, the lncRNA Smart Silencer was constructed by RiboBio, and the specific sequences are shown in **Table S4**. Approximately 24 h after cell transfection into two cancer cell lines (T24 and J82), the effect of overexpression or knockdown was detected using qPCR.

**Cell proliferation, colony formation, cell Transwell, and apoptosis assays**

For the cell proliferation assay, the transfected cells were cultured with complete medium and seeded in 96-well plates at a density of 5,000 cells/well. The cell numbers were quantified using a CCK-8 Kit (Dojindo) at 8 h, 24 h, 48 h, and 72 h. For the colony formation assay, the resuspended cells were placed in 6-well plates containing complete medium at a density of 1000 cells/well. After approximately 10 days of incubation, the cells were fixed with paraformaldehyde and stained with crystal violet (Beyotime). The number of cell colonies was counted under a microscope. For cell migration and invasion analysis, cells were resuspended in serum-free medium (3.0×10^4^ cells/well for migration and 6.0×10^4^ cells/well for invasion) and placed in Transwell inserts (Millipore) with or without Matrigel (BD Biosciences) in a 24-well plate containing complete medium in the lower chamber. After incubation for 24 h or 48 h, invaded or migrated cells were fixed with paraformaldehyde, stained with crystal violet (Beyotime) and counted under a microscope. For cell apoptosis, 24 h after cell transfection, the cells were resuspended in 100 μl of Buffer and incubated with FITC Annexin V (Vazyme) and PI (Vazyme) for 15 min in the dark, and the proportion of apoptotic cells was measured on a flow cytometer (Becton Dickinson). All experiments were performed in triplicate.

**URLs**

dbSNP, http://hgdownload.soe.ucsc.edu

RegulomeDB, http://www.regulomedb.org/

HeploRegv4.1, http://pubs.broadinstitute.org/mammals/haploreg/haploreg.php

SNPinfo Web Server, http://snpinfo.niehs.nih.gov/snpinfo/snpfunc.html

CancerSpliceQTL Database, http://www.cancersplicingqtl-hust.com

Ensembl, http://grch37.ensembl.org/index.html

GTEx, https://gtexportal.org/home/

CPAT, http://lilab.research.bcm.edu/cpat/

CPC2.0, http://cpc2.gao-lab.org/index.php

TCGA, http://cancergenome.nih.gov/

QTL base, http://www.mulinlab.org/qtlbase/index.html

VannoPortal, http://www.mulinlab.org/vportal/index.html

HPA, https://www.proteinatlas.org/

CCLE, https://sites.broadinstitute.org/ccle

3DSNP, http://cbportal.org/3dsnp/

GEPIA2.0, http://gepia2.cancer-pku.cn/#index

LncAS2Cancer, https://lncrna2as.cd120.com/

ncRNA-eQTL, http://ibi.hzau.edu.cn/ncRNA-eQTL/index.php

**Supplementary Figure 1.** The nuclear and cytoplasmic localization of *BCLET* in bladder cancer cells.

**
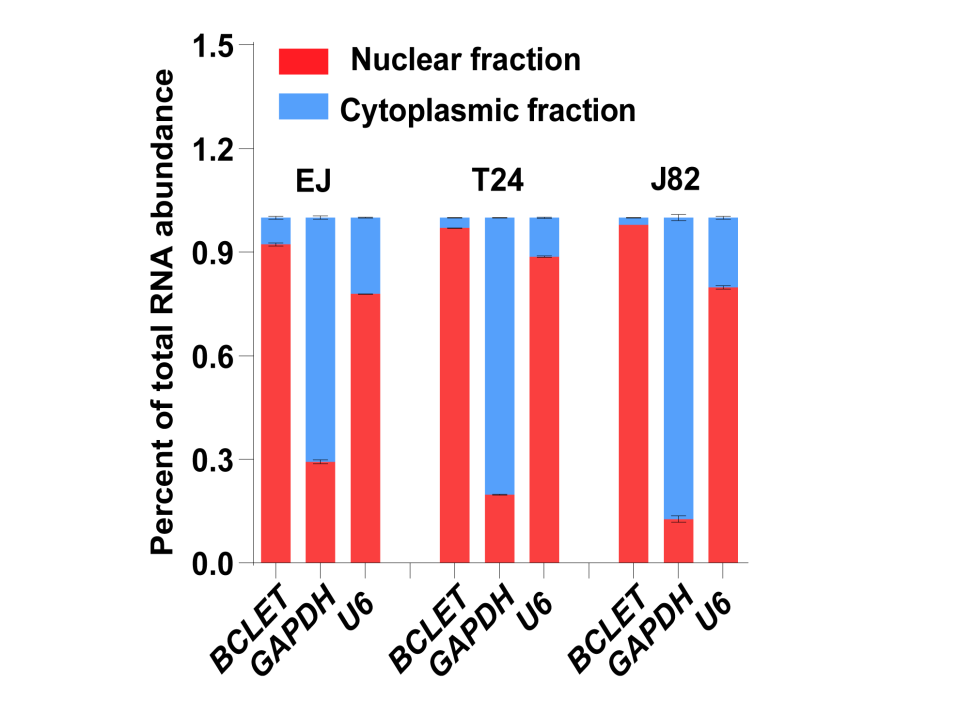
**

**Supplementary Figure 2.** Overexpression of *BCLET* transcripts in bladder cancer cells. (A) *BCLET*-long. (B) *BCLET*-short.


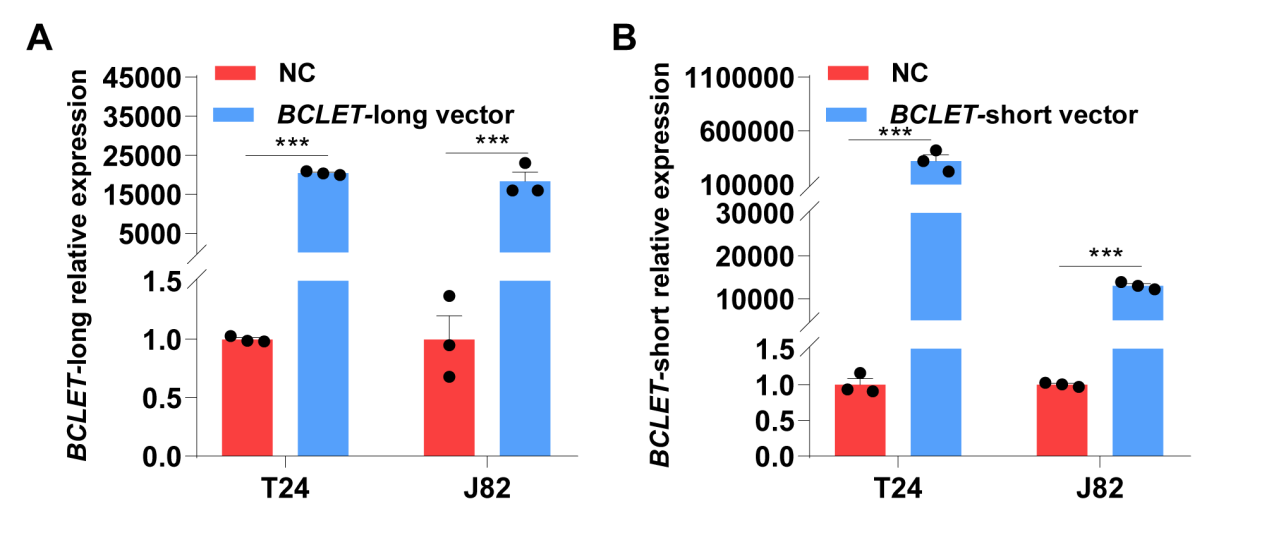


**Supplementary Figure 3*.*** *BCLET* transcripts significantly inhibited proliferation in J82 cell lines. The effect of *BCLET* transcripts overexpression on cell viability was detected by CCK-8 (A) and colony formation assays (B).


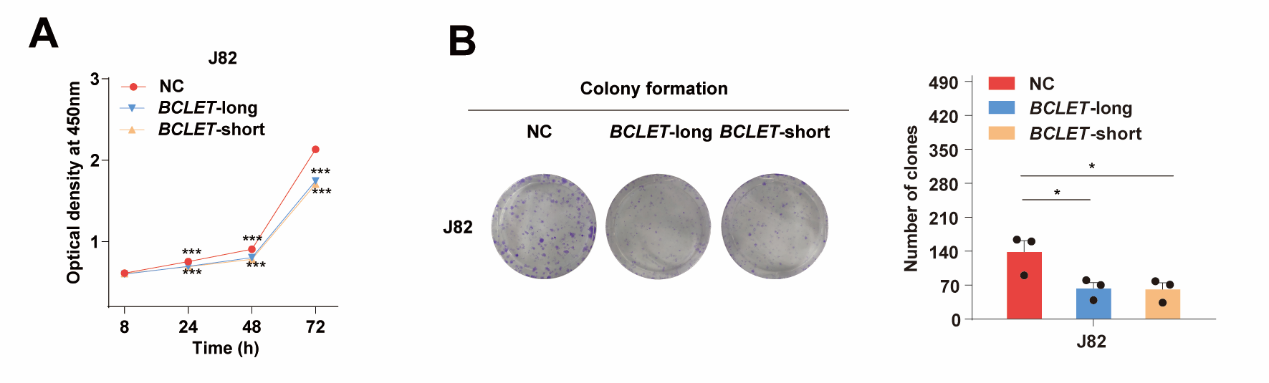


**Supplementary Figure 4*.*** *BCLET* transcripts significantly inhibited cell metastasis and promoted cell apoptosis. The effect of *BCLET* transcripts overexpression on bladder cancer cell migration (A) and invasion (B). (C) The effect of *BCLET* transcripts overexpression on cell apoptosis was assessed by flow cytometry.


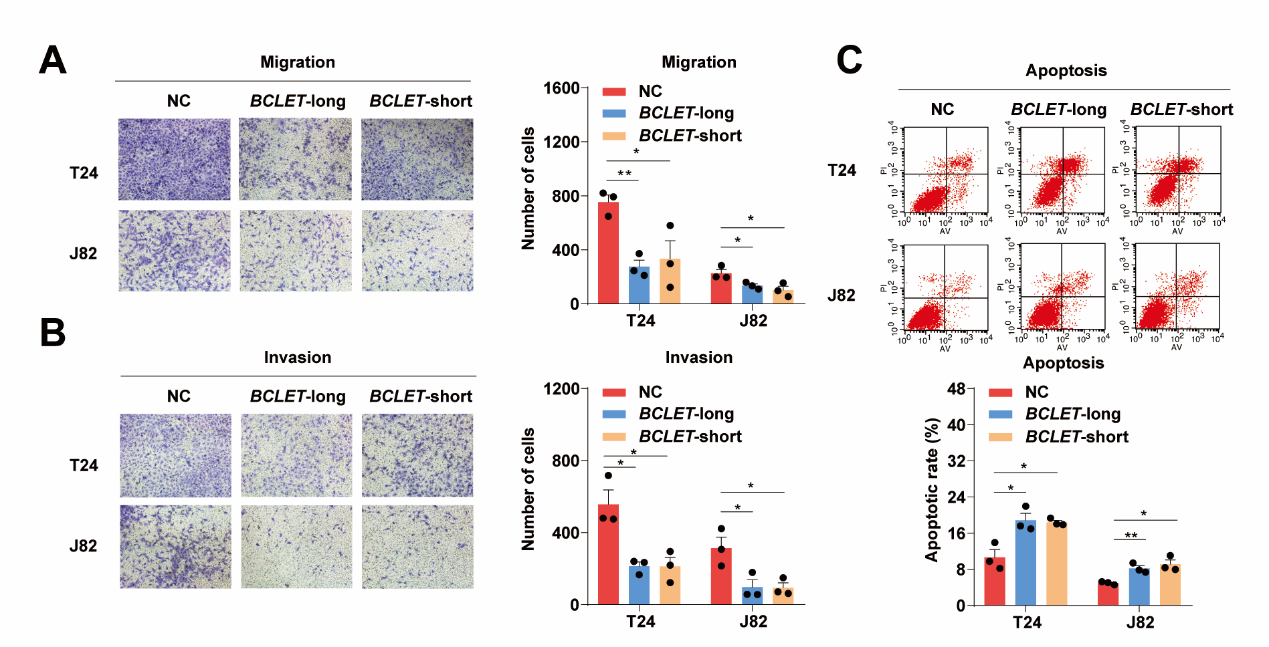


**Supplementary Figure 5.** Overexpression of *BCLET* can inhibit bladder carcinogenesis. *BCLET-*long/*BCLET*-short/NC lentiviral vector, namely LV-*BCLET-*long, LV-*BCLET*-short and LV-NC, were stably transfected into T24 cells. (A) Efficiency of stable overexpression of *BCLET*-long and *BCLET*-short transcripts, as detected using qRT-PCR. (B) cell proliferation. (C) colony-forming assays. (D) invasion and migration. **
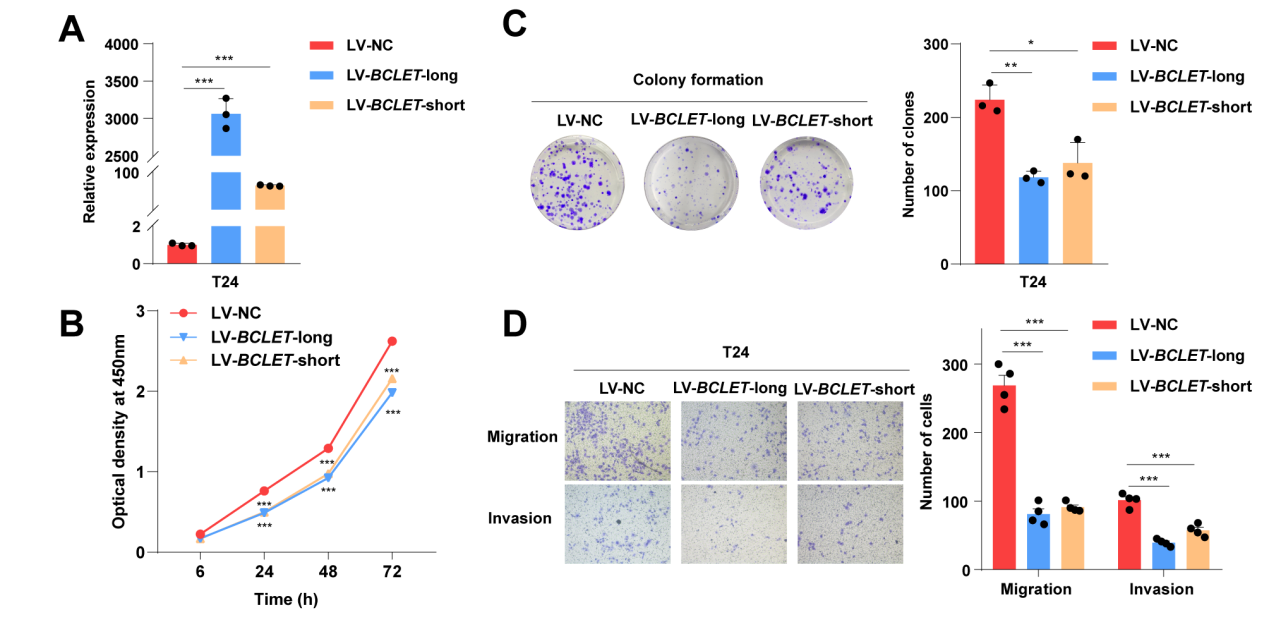
**

**Supplementary Figure 6.** Effect of knockdown of *BCLET* in bladder cancer cells.


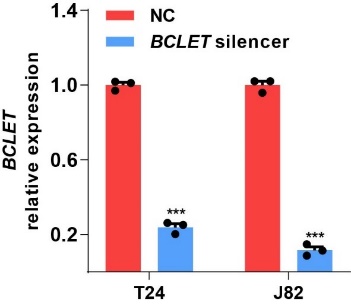


**Supplementary Figure 7.** Effect of overexpression of *MSANTD2-004* in bladder cancer cells.


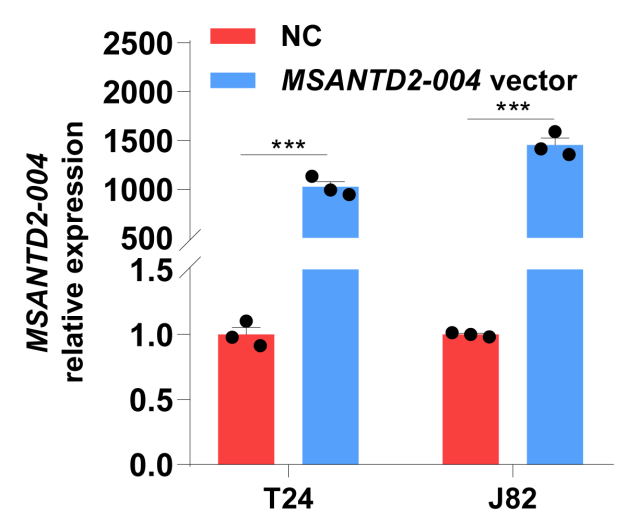


**Table S1.** The demographic characteristics of bladder case-control study

| **Variables** | **Discovery stage** | | | | | | |  | **Validation stage** | | | | | | |
| --- | --- | --- | --- | --- | --- | --- | --- | --- | --- | --- | --- | --- | --- | --- | --- |
|  | **Cases**  **(n = 580)** | |  | **Controls**  **(n = 1,101)** | |  | ***P*^a^** |  | **Cases**  **(n = 1,050)** | |  | **Controls**  **(n = 1,403)** | |  | ***P*^a^** |
|  | **N** | **%** |  | **N** | **%** |  |  |  | **N** | **%** |  | **N** | **%** |  |  |
| Age (mean±SD) | 64.7 ± 12.1 | |  | 64.5 ± 12.1 | |  | 0.689 |  | 64.8 ± 12.7 | |  | 65.2 ± 9.3 | |  | 0.320 |
| Sex |  |  |  |  |  |  |  |  |  |  |  |  |  |  |  |
| Male | 481 | 82.9 |  | 905 | 82.2 |  | 0.707 |  | 839 | 79.9 |  | 1,107 | 78.9 |  | 0.544 |
| Female | 99 | 17.1 |  | 196 | 17.8 |  |  |  | 211 | 20.1 |  | 296 | 21.1 |  |  |
| Smoking status |  |  |  |  |  |  |  |  |  |  |  |  |  |  |  |
| Never | 313 | 54.0 |  | 717 | 65.1 |  | < 0.001 |  | 533 | 52.7 |  | 866 | 61.7 |  | < 0.001 |
| Ever | 264 | 45.5 |  | 384 | 34.9 |  |  |  | 497 | 47.3 |  | 537 | 38.3 |  |  |
| Unknown | 3 | 0.5 |  | 0 | 0 |  |  |  |  |  |  |  |  |  |  |
| Grade |  |  |  |  |  |  |  |  |  |  |  |  |  |  |  |
| Low | 287 | 49.5 |  |  |  |  |  |  | 517 | 49.2 |  |  |  |  |  |
| Intermediate | 174 | 30.0 |  |  |  |  |  |  | 370 | 35.3 |  |  |  |  |  |
| High | 86 | 14.8 |  |  |  |  |  |  | 163 | 15.5 |  |  |  |  |  |
| Other^b^ | 33 | 5.7 |  |  |  |  |  |  |  |  |  |  |  |  |  |
| Stage |  |  |  |  |  |  |  |  |  |  |  |  |  |  |  |
| Non-muscle invasive | 377 | 65.0 |  |  |  |  |  |  | 688 | 65.5 |  |  |  |  |  |
| Invasive | 152 | 26.2 |  |  |  |  |  |  | 362 | 34.5 |  |  |  |  |  |
| Other | 51 | 8.8 |  |  |  |  |  |  |  |  |  |  |  |  |  |

^a^Two-sided *t* test or χ^2^ test.

^b^Other includes papilloma and missing data.

**Table S2.** Association between 9 candidate AS-SNPs and bladder cancer risk in the discovery stage

| **SNP** | **Chr** | **Position** | **Allele^a^** | **Gene** | **MAF** | | ***P*_HWE_** | **Call rate** | **OR (95% CI)** | ***P*^b^** |
| --- | --- | --- | --- | --- | --- | --- | --- | --- | --- | --- |
|  |  |  |  |  | **Cases** | **Controls** |  |  |  |  |
| rs558814 | 11 | 124675214 | A/G | *RP11-677M14.7* | 0.28 | 0.34 | 0.09 | 0.96 | 0.78 (0.67-0.91) | 1.91×10^-3^ |
| rs1877022 | 11 | 41842944 | G/C | *RP11-375D13.2* | 0.07 | 0.09 | 0.21 | 1.00 | 0.72 (0.55-0.95) | 1.88×10^-2^ |
| rs7220814 | 17 | 7290695 | A/G | *TNK1* | 0.19 | 0.22 | 1.00 | 1.00 | 0.81 (038-0397) | 2.33×10^-2^ |
| rs28359631 | 1 | 230898397 | A/G | *RP11-99J16_A.2* | 0.14 | 0.12 | 0.39 | 0.99 | 1.27 (1.02-1.57) | 3.13×10^-2^ |
| rs2075276 | 22 | 21363744 | T/C | *THAP7-AS1* | 0.12 | 0.15 | 0.12 | 0.98 | 0.79 (0.64-0.98) | 3.27×10^-2^ |
| rs11176575 | 12 | 40820208 | G/A | *RP11-115F18.1* | 0.42 | 0.46 | 0.86 | 0.99 | 0.85 (0.74-0.99) | 3.37×10^-2^ |
| rs496797 | 11 | 94225807 | C/T | *MRE11A* | 0.47 | 0.50 | 0.72 | 1.00 | 0.86 (0.75-0.99) | 4.34×10^-2^ |
| rs2100431 | 15 | 60770850 | C/A | *NARG2* | 0.07 | 0.06 | 0.15 | 1.00 | 1.33 (1.01-1.77) | 4.41×10^-2^ |
| rs7157977 | 14 | 31858209 | C/T | *HEATR5A* | 0.33 | 0.36 | 0.60 | 1.00 | 0.85 (0.74-0.99) | 4.66×10^-2^ |

SNP, single nucleotide polymorphism; MAF, minor allele frequency; HWE, Hardy-Weinberg equilibrium; OR, odds ratio; CI, confidence interval.

^a^Major/Minor.

^b^*P* values were calculated from logistic regression analysis adjusted for age and sex.

**Table S3.** Association between SNP rs558814 and bladder cancer risk in the validation stage

| **Genetic model** | **Genotypes** | **Cases** | |  | **Controls** | | **Adjusted OR (95% CI)** | ***P*^a^** |
| --- | --- | --- | --- | --- | --- | --- | --- | --- |
|  |  | **N** | **%** |  | **N** | **%** |  |  |
| Additive | A | 1,440 | 69.2 |  | 1,859 | 66.3 | 1.00 |  |
|  | G | 640 | 30.8 |  | 943 | 33.7 | 0.88 (0.78-0.99) | 0.033 |
| Codominant | AA | 502 | 48.3 |  | 617 | 44.0 | 1.00 |  |
|  | AG | 436 | 41.9 |  | 625 | 44.6 | 0.86 (0.72-1.02) | 0.075 |
|  | GG | 102 | 9.8 |  | 159 | 11.4 | 0.79 (0.60-1.04) | 0.088 |
| Dominant | AA | 502 | 48.3 |  | 617 | 44.0 | 1.00 |  |
|  | AG/GG | 538 | 51.7 |  | 784 | 56.0 | 0.84 (0.72-0.99) | 0.037 |
| Recessive | AA/AG | 938 | 90.2 |  | 1,242 | 88.7 | 1.00 |  |
|  | GG | 102 | 9.8 |  | 159 | 11.4 | 0.85 (0.65-1.10) | 0.219 |

OR, odds ratio; CI, confidence interval.

^a^*P* values were calculated from logistic regression analysis adjusted for age and sex.

**Table S4.** Sequences of primers and probes used in this study

| **Experiments** | **SNP/Gene** | **Description** | **Sequences (5'-3')** |
| --- | --- | --- | --- |
| TaqMan | rs558814 | Forward | TTTGAAGCCGAGGACTTTGTC |
|  |  | Reverse | GCCTGCCTGGCATTCTTCTA |
|  |  | P-G | FAM-TTCACTCCCAATGGA-MGB |
|  |  | P-A | HEX-ACTCCCAATAGATTCA-MGB |
| RT-PCR | *BCLET* | Forward | GGTAGGTGTGGCCGTTTGTA |
|  |  | Reverse | GCAACTTCCAAAGCACGGAG |
|  | *BCLET*-short | Forward | AGTAGCACAGCACATCCAGT |
|  |  | Reverse | TTGCATTCATTGGTCAGCATCC |
|  | *MSANTD2* | Forward | ATTCACAGGAGGACTGGGGAA |
|  |  | Reverse | TGCATGATGTCTCTCTTCTGTG |
|  | *MSANTD2*-*004* | Forward | ATCTGTGGTCCGTACCTGGA |
|  |  | Reverse | CCCATCCTCCCTGACCAAAC |
|  | *GAPDH* | Forward | CCGGGAAACTGTGGCGTGATGG |
|  |  | Reverse | AGGTGGAGGAGTGGGTGTCGCTGTT |
|  | *U6* | Forward | CTCGCTTCGGCAGCACA  AACGCTTCACGAATTTGCGT |
|  |  | Reverse | AACGCTTCACGAATTTGCGT |
| RIP | *MSANTD2-RIP* | Forward | GCTGGACAGACTTTTCAAGGC |
|  |  | Reverse | AGTACTCCCGAAGTCGCTTG |
| LncRNA Smart Silencer | *BCLET* |  | CAATCAAGGACAGTAAACA |
|  |  |  | CAACCAAGATGGAATAACA |
|  |  |  | CCAGCAGAAAAGTTACTTT |
|  |  |  | GAGAATTAATACAGGCTCCA |
|  |  |  | ACCAGCTGACGACAACCATA |
|  |  |  | TCCAATACCAGTCTCCATCC |
